# Gastric Cancer in Aktobe Region of Western Kazakhstan from 2009 to 2018: Incidence Rates, Trends, and Five-Year Survival

**DOI:** 10.31557/APJCP.2020.21.6.1645

**Published:** 2020-06

**Authors:** Saule K Balmagambetova, Yerbol Z Bekmukhambetov, Anar B Tulyaeva, Yerbolat M Iztleuov, Gaziza A Smagulova, Arip K Koyshybaev, Olzhas N Urazayev, Saganaj T Djussembekov, Valeriy V Begunov, Irakli Kokhreidze

**Affiliations:** 1 *West Kazakhstan Marat Ospanov Medical University, 68, Maresyev Street, Aktobe, Kazakhstan. *; 2 *Tbilisi State Medical University, 33, Vazha-Pshavela Ave., Tbilisi, Georgia. *

**Keywords:** Gastric cancer, incidence, trends, five-year survival, western Kazakhstan

## Abstract

**Objective:**

to assess the current state of gastric cancer (GC) incidence and its five-year survival across Aktobe region of western Kazakhstan from 2009 to 2018 by presenting key indicators and analyzing the most significant features.

**Methods:**

Rough incidence rates (per 100,000) and average annual percent changes (aAPCs) were estimated for each age group at diagnosis with respect to gender, ethnicity, residence, the disease stages, tumor subsite, and histology type using linear regression analysis, including the prognostic index for 2019-2020. Overall five-year survival rates were estimated by the Kaplan-Meier method.

**Results:**

Overall GC incidence increased from 19.2 to 29.3, and averaged 25.8 (R2 0.65) with aAPC of 3.2%, with a potential to further rise (30.4 by 2020, p**<**0.001). Non-cardia location (17.8, p**<**0.001, aAPC 6.4%) and intestinal type of the tumor (17.0, p**<**0.001, aAPC 7.35%) were predominant. The observed overall five-year survival rate was 28.4% (95% CI 24.5;32.3) with a median survival time of 8.0 months (95% CI 6.6;9.4). Groups aged 40-49 and ≥70 had the lowest rates (24.4% and 22.1%, respectively, log-rank p 0.008), but the youngest individuals (18-39 years) showed the shortest median survival time, 5.0 months after diagnosis at the survival rate of 29.4%. Resectional surgery contributed significantly to the median survival time, 23.0 months vs. 6.0 in non-operated patients (log-rank p**<**0.001).

**Conclusion:**

GC in Aktobe region was featured by growing incidence and unsatisfactory five-year survival rates. Indigenous males of 60-69 years old with intestinal histology type, as well as the youngest patients irrespective of their gender, ethnicity, and other characteristics were recognized as high risk groups. Besides, relatively high aAPC 5.1% in the youngest revealed their further expected vulnerability. Further research is suggested to focus on risk factors, including gene expression profiling, to find out an accessible preventive strategy.

## Introduction

According to the International Agency for Research on Cancer (IARC), Gastric cancer (GC) remains one of the most deadly neoplasms worldwide, particularly among older males. It is the fifth most common malignancy and the third leading cause of death in both sexes after lung cancer and colorectal cancer (Bray et al., 2018). Since gastric cancer has a multifactorial nature and it is one of the most behaviorally influenced cancers, its incidence is highly variable by region and culture (Rawla and Barsouk, 2019). In Eastern and Central Asia and Latin America, the highest incidence rates of GC are recorded (Balakrishnan et al., 2017). Researchers from Brazil also pointed out the highest GC incidence in Belem, 69.1/100,000 in men (2003) and 26.7/100,000 in women (2006) (Curado et al., 2019). The Republic of Korea has reported the highest national incidence of GC with almost 60 per 100,000 new cases annually for males (Ferlay et al., 2018). On the contrary, its overall incidence in the United States has been steadily declined over the past 75 years. The National Cancer Institute’s Surveillance, Epidemiology, and End Results (SEER)estimated that about 1/114 men and women will be diagnosed with GC during their life in 2009 (Lee et al., 2013). Meanwhile, this highly lethal tumor still is featured by relatively low five-year survival, within or less than 30% on a global scale (Crew and Neugut, 2006). 

The most predominant non-cardia cancers are brought about by chronic gastritis caused by *Helicobacter pilori* or other inflammation of the stomach lining which can be caused by a variety of environmental factors (Mukaisho et al., 2015). Along with this, it has been proven that the familial clustering of GC is seen in 10% of cases, and approximately 3% of cases arise in the setting of hereditary diffuse GC (HDGC). All reported HDGCs are pure diffuse type according to Lauren classification (Lauren, 1965) and are associated with dismal prognosis once the tumor invades the submucosa (Luo et al., 2018). In families with HDGC, GC is presented at a relatively young age before 40 years old. Germline mutations in the CDH1 gene are the major cause of HDGC and are identified in approximately 25-50% of families that fulfill strict CDH1 testing criteria (van der Post et al., 2015). Accordingly, countries with a high incidence of sporadic gastric cancer, such as Japan and Korea, have a lower frequency of germline mutations in familial GCs than low-incidence countries. The cause of familial clustering in high-incidence countries is more likely environmental than hereditary (Vogelaar et al., 2012). 

Within 2004-2013, the GC incidence rates in Kazakhstan fluctuated in frames of 21.8-25.6 per 100,000, with the peak rates in the age group of ≥70 and a proportion of 8.5% in the general structure of malignancies. Besides, a tendency in morbidity reduction was recorded between 2004 and 2013 (-18.0%), and the western region was referred to as the medium level zone of GC incidence (Orazova et al., 2015). 

Among the risk factors for GC development, environmental pollution is not being accented comparing to the role of *Helicobacter pilori*, while the Aktobe province of western Kazakhstan, as well as the entire western region, is known by its ecology troubles due to presence of oil, chromium producing, and other industries. Environmental pollution is also related to the fact that the Aktobe province is partially located in the vast Aral Sea ecological disaster region. According to data, long-time average annual levels of cancer morbidity for 2004-2013 in adult population living in the Aral Sea environs were up to 2.6 times higher, compared to ecologically intact terrains. In particular, long-time average annual level of cancer morbidity in adult population living in that zone was 57.2% higher, and the total cancer morbidity depended on the hazard coefficient (HQ) associated mostly with the inhalation of nickel and the combined cadmium intake (r=0.8) (Mamyrbayev et al., 2016). Overall, the Aktobe province is highly indicative of cancer morbidity due to the mentioned reasons. 

However, the analysis GC incidence rates and the disease’s main features, such as the distribution of histology types, proportion of tumor subsites and stages at diagnosis, dynamics across the age groups, etc., has not been performed by local researchers yet.

Thus, the present study aimed to assess the current state of GC incidence and its five-year survival across Aktobe province of the western region by presenting key indicators and analyzing the most significant features.

## Materials and Methods

*Study setting*

This retrospective database research was approved by the University’s IREC (Protocol No. 24, 24.05.2019) and performed following the Helsinki Declaration principles. Informed consent was not required as the individually identifiable data of patients were not involved. 

The present research scopes terrain of the Aktobe province, the largest in western Kazakhstan (Central Asia). Indigenous inhabitants are Kazakhs constituting 82.4% of the total population, of Turkic ethnic group, followed by Slavs and other ethnic groups (Azerbaijanians, Koreans, Tatars, Germans, etc.). Population density in western region is the smallest in the country, 3.4 per km^2^, according to data of the National Statistics Agency in 2016. 

*Study population*

Data on the province’s total adult population (≥18) from 2009 to2018 (522,282 in January 2009 and 587,544 in January 2018), including males/females, and by age groups were requested from the Aktobe Statistical Committee. 

All incident first diagnosed GC cases (C16.0-C.16.9, The WHO ICD-10 Version: 2016) from 2009 to 2018 in adults aged 18 years and older were obtained from the Cancer Registry of the Aktobe Regional Oncologic Center. Rough incidence rates (per 100,000) and average annual percent changes (aAPCs), as well as the ratios were estimated for each age group at diagnosis (18-39, 40-49, 50-59, 60-69, and ≥70 years), with respect to gender, ethnicities (Kazakhs and others), residence (urban or rural area), and the disease clinical stage (The 8^th^ edition of the UICC TNM classification, 2016: gastric carcinoma, adenocarcinoma). Stages were presented as St I; St II (St IIA + St IIB – T_1-4a_; N_0-3_, M_0_); St III; and St IV (T_4b_, any T; any N; M_0-1_).

Regarding the histology type, we matched definitions presented by Lauren’s and WHO classifications: “indeterminate” type by Lauren is defined as a mixed type by the WHO. “Adenocarcinoma with mixed subtypes, or Adenocarcinoma combined with other types of carcinoma” is encoded as 8255/3 in the WHO third edition of the International Classification of Diseases for Oncology (ICD-O), 2013 (Berlth et al., 2014). Some authors missed this histologic type referring to as the previous classifications, where the mixed type was the part of diffuse type, but in our Registry, the mixed type was recorded in the amount of 9.6%. Overall, all GC cases in the present paper were assigned into three histologic types: diffuse, intestinal, and mixed ones.

Categorization of cases by the sites was depended on the number of cases. Keeping in mind the fact that the number of C16.8 and C16.9 cases in our Registry was negligibly small (15 out of 1,458), we merged non-cardia sites (C16.1-C16.6) with codes 16.8–16.9. A tumor was coded 16.8 (overlapping lesion of the stomach), when the location of the lesion was in the anterior or posterior wall of the stomach and none of the specific sites was mentioned, and cases with code 16.9 (stomach, NOS) were cases with missing site codes. So, this research dealt with two main sublocations, namely C16.0 – cardia, C16.1-C16.9 – non-cardia, and other GC cases. The observed five-year survival rates were also stratified by age group, histology type, clinical stage, the tumor subsite, as well as resectional surgery.

*Data analysis*

Calculations were done in Statistica.10 (Dell Technologies, Round Rock, Texas, USA) as well as SPSS (version 25) (IBM, Armonk, USA). For all tests, a two-sided type I error of p<0.05 at 95% confidence interval (CI) was assumed statistically significant. Rough incidence rates and corresponding 95% CIs were determined by linear regression analysis and expressed as the number of cases per 100,000 individuals, including the prognostic index for 2019-2020. Changes in the incidence rates were presented as a summary measure of a trend over a fixed period of time and expressed as aAPCs. Five-year survival rates were estimated through commonly accepted Kaplan-Meier analysis by defining the differences between groups through the log-rank criterion. 

## Results

In the Aktobe Cancer Registry, a total of 1,458 records of patients who were first diagnosed with GC over the 2009-2018 period were found. Descriptive statistics of GC patients is presented in the first two columns of Table 1.

Kazakh men aged ≥ 60 with non-cardia location of tumor and predominance of intestinal histology type had the highest prevalence. Besides, most of them were at stage II or III at the time of diagnosis; whereas, cases registered with stage I had the lowest prevalence (4%). The residence of patients with GC did not have a significant effect on.

**Figure 1:**
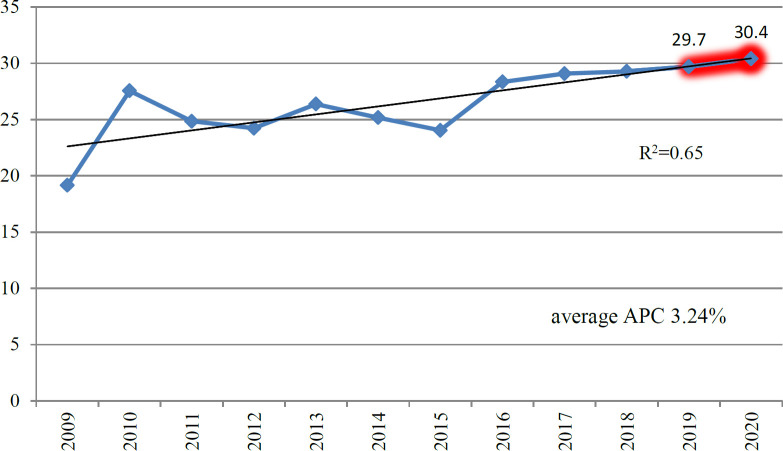
GC Incidence Over Years, Including Prognostic Indices for 2019-2020

Overall dynamics in incidence by year evidenced that an increase in GC rate from 19.2 in 2009 up to 29.3 in 2018 was featured by uneven spasmodic growth with a relative outbreak of unexplained nature in 2010 and a smooth increase starting since 2015. Such a pattern was more expressive in men compared to women. The proportion of stages I and II (resectable) was not increased for a decade as noticeably as advanced stages of III and IV (9.3 to 13.3 vs. 9.2 to 16.0, respectively). Non-cardia location prevalence increased almost twice (from 12.8 up to 22.3), unlike the cardia site of the tumor. Regarding the histology types, the prevalence of intestinal type also incremented from 11.9 to 19.9 over the period compared to diffuse and mixed ones. The most active dynamics was observed in age groups of 40-49 and 60-69 years old. Overall incidence rate dynamics is reflected in Figure 1, where prognostic indices for 2019-2020 are also presented. 

**Figure 2:**
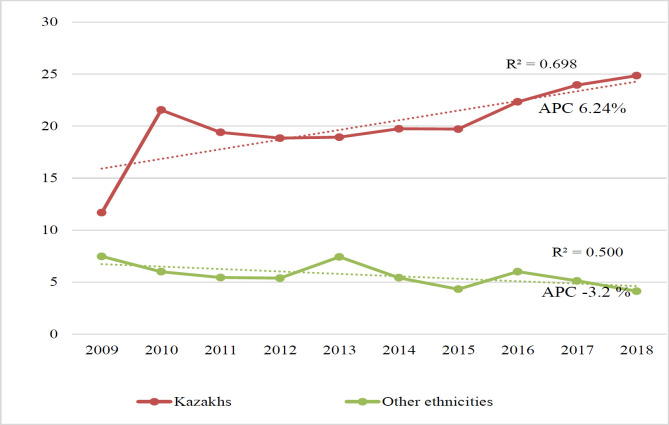
GC Incidence Across Various Ethnicities

Thus, further increase in GC incidence rate from 19.2 in 2009 up to 29.7 (95% CI 23.9; 32.7) in 2019 and to 30.4 (95% CI 26.9; 33.5) in 2020 is expected (R^2^ 0.65, p<0.001). It is noteworthy to mention that this outbreak of 2010 was observed in the graph on incidence dynamics among Kazakh patients only, unlike other ethnicities. Dynamics in incidence rates across various ethnicities is presented in Figure 2.

Table 1 also reflects overall data on the incidence by explored parameters and average aAPCs for different population groups. 

Quite remarkable is an expected decrease in morbidity among the other ethnicities, unlike the Kazakhs, where the trend stayed positive (aAPC -3.2 vs. 6.2%). Another negative trend, albeit non-significant, was noted in the cardia subsite of the tumor (aAPC -1.4%), where the proportion was 32.9% through the last decade. Regarding the age group, increasing trend in GC cases among patients aged 60-69 and relatively sharp growth in its incidence (aAPC 5.1%) among patients aged < 39 years old was expected. Generally, young individuals are usually being diagnosed hereditary diffuse GC, but the trend in diffuse GC was relatively small compared to intestinal one (aAPC 3.5 vs. 7.4%).

The observed five-year survival was calculated (N= 762 from 2014 to 2018) and stratified by age, tumor location, clinical stage, histology type, and resectional surgery. Overall five-year survival rate was calculated as 28.4% at the median survival time of 8 months. Obtained survival rates are summarized in Table 2 and in corresponding graphs. 

Survival rate depending on clinical stage consecutively declined from 50.5% at stage I to 7.3%. 

Though the incidence rate smallest values were observed in the age groups of 40-49 and ≤70 years old, shorter median survival time was revealed in the youngest patients (5 months after diagnosis vs. 11 and 6 months, respectively).

Non-cardia location was the more favorable for survival time than the cardia site of the tumor ( 11 months vs. 7 months).

Patients with the intestinal type of tumor showed a relatively large rate, 30.7%, as well as the survival time, 9 months, compared to other types. 

Surgical treatment significantly impacted the median survival time, being 23 months in those who underwent surgery vs. 6 months in non-operated patients. 

**Table 1.** Descriptive Characteristics of Population First Diagnosed with GC and the Rough Incidence Rates for All Studied Parameters and Population Groups, Including Trends

Population groups	N	%	Rate (un-adjusted)	St. error	95% CI, lower	95% CI, upper	Ratio	*P*-value	aAPC, %
Total	1,458	100	25.8	2.1	21.6	30			3.2
Gender:									
Females	490	33.6	8.7	1.2	6.3	11.1	ref.		3.1
Males	968	66.4	17.1	1.7	13.7	20.5	2	<0.001	3.2
Ethnicity:									
Others	299	20.5	5.7	0.4	4.9	6.5	ref.		-3.2
Kazakhs	1,159	79.5	20.1	1.1	17.5	22.7	3.9	< 0.05	6.2
Residence:									
Rural area	691	47.4	12.6	0.5	11.4	13.8	ref.		2.5
Urban area	767	52.6	13.2	0.6	11.8	14.7	1.1	0.39	4
Age									
18-39	60	4.1	0.9	0.1	0.7	1.3	ref.		5.1
40-49	133	9.1	2.2	0.2	1.7	2.7	2.2	<0.001	1.5
50-59	386	26.5	6.9	0.3	6.2	7.5	6.4	<0.001	2.5
60-69	440	30.2	7.8	0.5	6.8	8.9	7.3	<0.001	5.7
70+	439	30.1	7.9	0.4	7	8.8	7.3	<0.001	2.3
Stage:									
I	58	4	0.9	0.2	0.6	1.4	ref.		0.5
II	706	48.4	12.3	0.8	10.5	14.1	12.2	<0.001	4.5
III	491	33.7	8.7	0.6	7.3	10.1	8.5	<0.001	1.8
IV	203	14	3.8	0.3	3.2	4.4	3.5	<0.001	3.8
Tumor site:									
C16.0 (cardia) Non-cardia	480	32.9	7.9	0.5	6.8	9.1	ref.		-1.4
other GC sites (C16.1-C.16.9)	978	67.1	17.8	0.9	15.5	19.9	2	<0.001	6.4
Histology type:									
Mixed	140	9.6	2.6	0.3	1.8	3.3	ref.		4.3
Diffuse	402	27.6	6.2	0.8	4.3	8.1	2.9	0.061	3.5
Intestinal	916	62.8	17	1.3	14.1	20	6.5	<0.001	7.4

**Table 2:** Overall Five-Year Survival Rates with Respect to Age Group, Tumor Location, Histology Type, Clinical Stage, and Resectional Surgery (N =762).

Parameters	5-year survival %	St. error	95% CI	Median survival time, months	St. error	95% CI	Log-rank p
Overall survival	28.4	2.1	24.5;32.3	8	0.7	6.6;9.4	
By age groups							0.008
18-39 yrs	29.4	9	11.8;47.0	5	1.4	2.3;7.8	
40-49	24.4	5.9	12.8;36.0	11	2.6	5.9;16.1	
50-59	30.5	4.5	21.7;39.3	9	1.5	6.0;12.0	
60-69	33.9	3.7	26.7;41.2	11	1.7	7.6;14.4	
70+	22.1	3.1	16.0;28.2	6	0.6	4.8;7.2	
By tumor location							0.18
Cardia	28.5	2.2	24.2;32.8	7	0.6	5.8;8.2	
Non-cardia and other cases	30.2	3.5	23.3;37.1	11	1.5	8.2;13.8	
By tumor morphology							0.047
Diffuse type	25.9	3.7	18.6;33.2	7	0.9	5.2;8.8	
Intestinal type	30.7	2.6	25.6;35.8	9	1	6.9;11.0	
Mixed type	19.5	5.3	9.1;29.9	7	1.2	4.6;9.4	
By stages							<0.001
I	50.5	15.7	11.9;79.1	-			
II	25.4	4.1	17.4;33.4	12	1.3	9.4;14.5	
III	22.4	3.8	15.0;29.8	9	1.8	5.4;12.6	
IV	7.3	3.4	0.6;14.0	4	0.6	2.9;5.1	
By presence/absence of surgical treatment							<0.001
Undergone surgery	15.3	9.1	3.1;37.0	23	3.9	15.2;30.8	
Non-operated	13.2	2.6	8.1;18.3	6	0.5	4.9;7.0	

**Figure 3:**
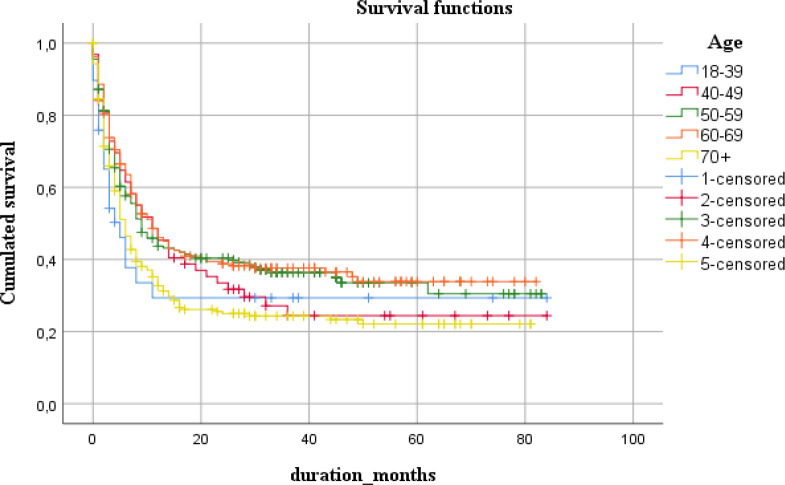
Five-Year Survival Across Different Age Groups

**Figure 4:**
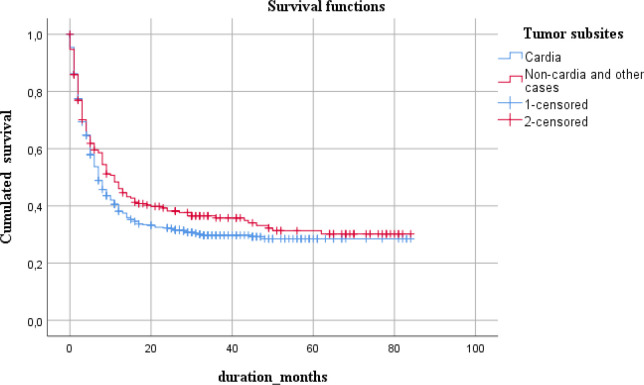
Five-Year Survival with Respect to Tumor Subsites

**Figure 5:**
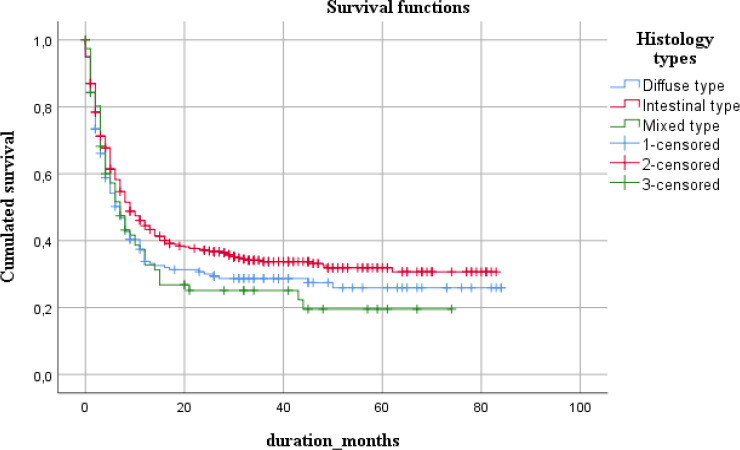
Five-Year survival with Respect to Tumor Morphology

**Figure 6:**
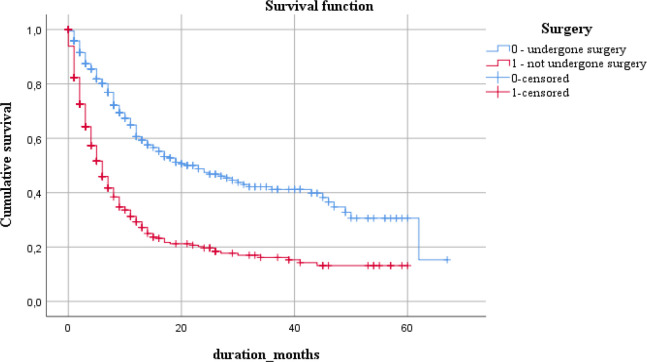
Five-Year Survival in Terms of Presence or Absence of Surgical Treatment

## Discussion

Relatively quick growth in the GC incidence rates from 19.2 up to 29.3 in 2018 observed in the Aktobe region is similar to data obtained in the borderline Uralsk region for almost the same period reporting 22.5 to 27.5 in men and 8.5 to 16.0 in women (Umarova et al., 2016). Given that the incidence is incremented mostly among the indigenous population, this phenomenon calls for some reasonable explanation. Overall, there are globally evidenced racial, ethnic, and gender disparities in the GC incidence (Nagini, 2015; Luo et al., 2017). For instance, between 1988 and 2012 in California, Korean Americans had about five times greater incidence of GC than Non-Hispanic Whites and twice that of Japanese Americans (Lee et al., 2017). Similar disparities were also observed in our analysis detecting that men/women ratio was 2:1 and Kazakhs/other ethnic groups ratio was 5:1. One of the reasons for ethnic disparities in GC might have been due to relatively fast transition into western nutrition style with a wide consuming of ultra-processed food; whereas, the traditional diet of Kazakhstan was of nomadic nature and mostly consisted of boiled meat and dairy. Meanwhile, a large number of research has proved that even a 10% increase in the proportion of ultra-processed food in the diet is associated with an increase of more than 10% in the risks of overall cancer, not counting other various consequences, such as metabolic syndrome or diabetes mellitus (Fiolet et al., 2018; GBD Diet Collaborators, 2019; Hall et al., 2019). 

Obvious disparities between GC patients from East and West were described by Griniatsos and Trafalis in 2018. According to them, GCs in the East are mainly diagnosed at younger ages, they are of intestinal type, and diagnosed at an early stage of the disease, while GCs in the West are mainly affecting elderly patients, they are of diffuse type, and diagnosed at an advanced stage. In our analysis, we reported prevalence of the age group of 60-69 (30.2%), intestinal histotype (62.8%), and non-cardia location of the tumor (67.1%), but we could not present the proportion of histology types and the tumor subsites by ethnic groups and their distribution by age. The mentioned researchers (Griniatsos and Trafalis) analyzed data from the US NCI’s SEER registries (N 13,840) and reported the median overall survival of 6 months in patients aged ≤ 44 years old compared to 3 months in patients aged 75 years old and older. Similarly, our analysis revealed quite similar data on median survival, revealing median survival of 5 months in those aged 18-39 years old and 6 months in patients aged ≥70. 

Based on the US SEER data, another study confirmed the racial disparities in the GC key indices in Asian and Caucasian patients. Although it reported significantly high median survival time among patients with IB, IIA, or IIB disease, the Asian patients had 37, 72, and 13 months longer median survival time than the corresponding Caucasian patients (Wang et al., 2015).

We obtained relatively similar survival rates in those underwent surgery and those who did not undergo surgery. 15.3% and 13.2%, with corresponding survival time 23 months and 6, respectively. Researchers from Sweden reported that the relative postoperative 5-year survival increased up to 43-44%; whereas, it stayed within 2-5% in non-operated patients for the last two decades (Asplund et al., 2018). 

In general, the five-year survival rate is relatively good only in Japan, where it reaches 90%. In European countries, survival rate varies from ~10% to 30%. High survival rate in Japan is probably achieved by early diagnosis using endoscopic examinations and consecutive early tumor resection (Sitarz et al., 2018).

To our knowledge, there are a few prevention strategies to minimize GC lethality. *Helicobacter pylori* eradication therapy significantly reduced GC incidence by 39%, as it was shown in the extended Shandong trial that lasted 14.3 years. Consensus groups from Asia, Europe, and Japan have recommended *H. pylori* eradication as primary prevention in high-risk areas. Following eradication therapy, endoscopic surveillance of pre-malignant lesions using enhanced imaging appears to be another effective preventive strategy (Fock, 2014). Pre-endoscopic risk assessment should be based on demographic and clinical features, such as ethnicity, age, gender, smoking, and *H. pylori* status. Nevertheless, just a few Asian countries with a high risk of GC have been applied population-based screening programs for daily practice (Quach et al., 2019). Another prospective way to prevent growing incidence GCof is the implementation of genetic counseling in relatives of patients with revealed of GC symptoms at an early age and suspected for hereditary diffuse GC (Moslim et al., 2018). 

Overall, GC in the Aktobe region currently is featured by growing incidence and unsatisfactory five-year survival rate. Indigenous males aged 60-69 years old with intestinal histology type and the youngest patients irrespective their gender, ethnicity, and other characteristics are high risk groups. Besides, relatively high aAPC of 5.1% in the youngest reveals their further expected vulnerability. 

Though the study introduced the current pattern of GC in western Kazakhstan, it had a number of limitations. One of the limitations was related to comparative shortness in analyzing the survival rate. Besides, it would be logically justified to present also the data on mortality through the region. 

Low rates of the five-year survival, particularly among the youngest, call for an in-depth analysis of the treatment and prevention tactics practiced by health providers in the region. 

Further research is suggested to focus on risk factors, including gene expression profiling, to find out an accessible preventive strategy.
